# Occupational Injuries in Germany: Population-Wide National Survey Data Emphasize the Importance of Work-Related Factors

**DOI:** 10.1371/journal.pone.0148798

**Published:** 2016-02-09

**Authors:** Alexander Rommel, Gianni Varnaccia, Nils Lahmann, Jan Kottner, Lars Eric Kroll

**Affiliations:** 1 Robert Koch Institute, Epidemiology and Health Monitoring, General-Pape-Str. 62–66, 12101, Berlin, Germany; 2 Charité-Universitätsmedizin Berlin, Institute of Health Sciences Education and Nursing, Augustenburger Platz 1, 13353, Berlin, Germany; 3 Charité-Universitätsmedizin Berlin, Clinical Research Center for Hair and Skin Science, Charitéplatz 1, 10117, Berlin, Germany; Leibniz Institute for Prevention Research and Epidemiology (BIPS), GERMANY

## Abstract

Unintentional injuries cause much of the global mortality burden, with the workplace being a common accident setting. Even in high-income economies, occupational injury figures remain remarkably high. Because risk factors for occupational injuries are prone to confounding, the present research takes a comprehensive approach. To better understand the occurrence of occupational injuries, sociodemographic factors and work- and health-related factors are tested simultaneously. Thus, the present analysis aims to develop a comprehensive epidemiological model that facilitates the explanation of varying injury rates in the workplace. The representative phone survey German Health Update 2010 provides information on medically treated occupational injuries sustained in the year prior to the interview. Data were collected on sociodemographics, occupation, working conditions, health-related behaviors, and chronic diseases. For the economically active population (18–70 years, n = 14,041), the 12-month prevalence of occupational injuries was calculated with a 95% confidence interval (CI). Blockwise multiple logistic regression was applied to successively include different groups of variables. Overall, 2.8% (95% CI 2.4–3.2) of the gainfully employed population report at least one occupational injury (women: 0.9%; 95% CI 0.7–1.2; men: 4.3%; 95% CI 3.7–5.0). In the fully adjusted model, male gender (OR 3.16) and age 18–29 (OR 1.54), as well as agricultural (OR 5.40), technical (OR 3.41), skilled service (OR 4.24) or manual (OR 5.12), and unskilled service (OR 3.13) or manual (OR 4.97) occupations are associated with higher chances of occupational injuries. The same holds for frequent stressors such as heavy carrying (OR 1.78), working in awkward postures (OR 1.46), environmental stress (OR 1.48), and working under pressure (OR 1.41). Among health-related variables, physical inactivity (OR 1.47) and obesity (OR 1.73) present a significantly higher chance of occupational injuries. While the odds for most work-related factors were as expected, the associations for health-related factors such as smoking, drinking, and chronic diseases were rather weak. In part, this may be due to context-specific factors such as safety and workplace regulations in high-income countries like Germany. This assumption could guide further research, taking a multi-level approach to international comparisons.

## Introduction

Injuries are a main cause of death, and lead to a considerable reduction in healthy life years. According to the World Health Organization’s Global Burden of Disease study, there are about 5 million injury-related deaths annually, accounting for nearly 10% of the global mortality burden [1]. The majority of all fatal injuries (3.9 million annually) are unintentional, leading to an annual loss of 138 million disability-adjusted life years [2].

The workplace is one of the main injury settings: worldwide, there are 313 million nonfatal occupational injuries each year requiring at least 4 days of absence from work. Annually, over 350,000 people die from occupational injuries [3]. As a result of improved safety regulations and a shift in economic activities from the industrial to the service sector, high-income countries bear a lower burden of occupational injuries [4]. Nevertheless, occupational injuries are still of concern in the developed world. In the United States, more than 3 million nonfatal occupational injuries are reported annually by private-sector employers [5]. A rather conservative estimate for the European Union gives a similar figure for both the private and the public sectors [6]. Germany alone registers about 1 million notifiable occupational injuries per year, accounting for 13% of all nonfatal injuries and resulting in 7% of the country’s annual sick leave [7, 8].

Epidemiological research on occupational injuries is usually focused on individual-level characteristics and emphasizes determinants that may either increase or decrease the risk of injury [9]. In previous studies, a wide range of such determinants has been identified. The dominant work-related risk factor for occupational injuries is the type of occupation, especially when it involves manual tasks or little vocational training. Other work-related risk factors include stand-alone indicators like limited work experience, but above all a wide range of specific working conditions. The latter can be roughly divided into the two dimensions of psychosocial (e.g. shift work, overtime, bad working climate) and physical stress (e.g. physically demanding tasks or workplace environments) [9–25]. The extent to which non-work-related factors may increase the risk of occupational injuries is also discussed. Apart from sociodemographics (e.g. young age, male gender) these include health-related risk factors such as smoking [10, 16, 18, 26–30], physical inactivity [14, 31], alcohol consumption [10, 18, 30, 32–34], and obesity [30, 31, 35–37], as well as prior medical conditions such as cardiovascular disease [14, 38–40], mental illness [11, 32, 38, 40], musculoskeletal conditions [18, 38, 39], diabetes [38–42], and asthma [38, 40].

However, when examining the potential effects of these factors, one should consider that the three main dimensions–occupation, working conditions (physical and psychosocial), and health-related factors–do not independently affect the chances of suffering occupational injuries. For instance, the association between health-related risk behaviors and occupational injuries is confounded by the socioeconomic status that is inherent in a person’s occupation. Because smoking and physical inactivity are more prevalent in lower social strata, where accident-prone occupations prevail, the effect of health-related risk behaviors on occupational injuries can hardly be estimated without accounting for socioeconomic factors such as the type of occupation.

Thus, the present analysis aims to develop a comprehensive epidemiological model that facilitates the explanation of varying injury rates in the workplace. In accordance with previous studies (see above), the underlying hypotheses imply that male gender, young age, less work experience, long regular working hours, a manual occupation, little vocational training, and work-related psychosocial and physical stress, as well as behavioral risk factors and chronic conditions, increase the chance of occupational injuries. Using Germany as an example of a high-income economy, it is the overall aim of the present study to identify the factors associated with an increased chance of self-reported occupational injuries. Provided that the type of occupation is one of the main indicators, this aim can be broken down into two more specific objectives: First, we examine whether physical stress and psychosocial stress are independently associated with the occurrence of occupational injuries. Second, we test the assumption that health-related factors may contribute to the explanation of occupational injuries when the type of occupation and work-related stress are taken into account.

## Materials and Methods

The German Health Update 2010 (GEDA 2010) is a representative phone survey of the German-speaking adult population in private households with a landline [43]. The sampling frame was a sample of phone numbers that forms the basis for most of the phone surveys in Germany. The Waksberg approach was applied to achieve a similar sampling probability for published and unpublished phone numbers [44, 45]. Random digit dialing was used to make contact with households. On the household level, the last-birthday method was applied to select the target persons. Computer-assisted telephone interviews (n = 22,050) were conducted by the Robert Koch Institute between September 2009 and July 2010. The cooperation rate on the individual level was 55.8%, which corresponds to the rates of other phone surveys in Germany and the United States [46, 47]. The average interview time was 31 minutes. The software VOXCO Interviewer Suite 5.4.4.5 was used for data collection. Interviewer performance was regularly supervised according to the Behavioral Risk Factor Surveillance System [48].

The German Health Update 2010 (GEDA 2010) is part of the Federal Health Monitoring which is a statutory task of the government-run Robert Koch Institute. GEDA 2010 was a general health survey designed to serve many data analyses [43]. The data analysis presented in this paper is not a separate study, as the participants were not contacted again. Interviews were done by specially trained in-house staff employed by the Robert Koch Institute, and not by external or commercial representatives. The authors of the present paper did not participate in the interviews. The combination of random digit dialing and the last-birthday method ensured that participants were selected without collecting participant identifying information. Phone numbers were deleted after completion of the interviews. Thus, at no time did either the authors or any other staff of the Robert Koch Institute have access to participant identifying information like names or addresses. Verbal informed consent was provided by all participants prior to the interview and was recorded electronically. After a data privacy statement was read to the participants, they had the opportunity to withdraw from the interview. As participation in GEDA 2010 was voluntary, at no cost to the survey participants, and because the study had no medical relevance for individual survey participants (no medical research involving human subjects is being conducted) ethics approval was not compulsory. Study design, methods of data collection, and consent procedure were approved by The Federal Commissioner for Data Protection and Freedom of Information [43].

An injury can be defined as: “A (suspected) bodily lesion resulting from acute overexposure to energy (…) interacting with the body in amounts or rates that exceed the threshold of physiological tolerance”. An occupational injury is an unintentional injury “(…) taking place during the performance of professional and paid activity (…)” [49]. In GEDA 2010, by means of a supplementary injury module, data were collected on up to three medically treated unintentional injuries that had occurred in the twelve months prior to the interview [50]. The main outcome was the declaration of at least one unintentional injury that the respondents assigned to the accident setting “at workplace.” Commuting accidents were not classified as occupational injuries.

To achieve the aims of this analysis, the factors that may be associated with the occurrence of occupational injuries were divided in four blocks of variables: (I) basic factors, (II) type of occupation, (III) indicators for work-related stress, and (IV) health-related factors. The basic factors included age, gender, work schedule, and years of employment. While a threefold age categorization was chosen for multivariate modeling (18–29, 30–49, 50+), a more detailed classification was used to give a clearer picture of descriptive age distributions (18–29, 30–39, 40–49, 50–59, 60+). The work schedule served to approximate different lengths of exposure to work-related hazards, and differentiates between part-time and full-time employees, and those who regularly work for more than 48 hours per week. Duration of employment in years was used as a proxy for work experience. The Blossfeld scheme, which was developed to fit the German labor market, served to distinguish 12 occupational groups (Table 1) [51].

**Table 1:** Sampling characteristics, gainfully employed men and women (age 18–70), GEDA 2010, n = 14,041.

	n	% (unweighted)	% (weighted)
**Sex**			
Women	7,522	53.6%	45.3%
**Age Group**			
18–29	2,634	18.8%	19.9%
30–39	2,944	21.0%	21.6%
40–49	4,375	31.2%	30.3%
50–59	3,121	22.2%	22.1%
60+	967	6.9%	6.1%
**Occupational injury**			
Yes	303	2.2%	2.8%
Missing	21		
**Work schedule**			
Full-time	7,181	51.4%	55.0%
Part-time.	4,787	34.2%	31.0%
> 48h/week	2,011	14.4%	13.9%
Missing	62		
**Occupational group**			
Skilled commercial and administrational occupations	2,769	20.2%	18.1%
Professions	741	5.4%	3.3%
Engineers	534	3.9%	2.8%
Managers	1,204	8.8%	6.8%
Unskilled commercial and administrational occupations	1,092	8.0%	8.7%
Semiprofessions	2,154	15.7%	11.8%
Technicians	598	4.4%	4.0%
Skilled services	1,294	9.4%	8.9%
Unskilled services	1,223	8.9%	12.8%
Skilled manual occupations	1,191	8.7%	11.9%
Unskilled manual occupations	687	5.0%	8.6%
Agricultural occupations	244	1.8%	2.3%
Missing	310		

Information on work-related stress was gathered using a concise module on typical exposures in the workplace, relying on well-established indicators [52, 53]. The module does not allow for the identification of concrete noxae, but helps to provide a rough overview of the employees’ working conditions. Physical stressors were defined as (I) lifting and carrying weights of more than 10 kg (below: heavy carrying), (II) working in squatting, stooping, kneeling, or lying postures or overhead (awkward postures), and (III) a working environment characterized by noise, cold, heat, dirt, smoke, or unfavorable illumination (environmental stress). Moreover, the following psychosocial stressors were considered: (I) a disturbed working climate, conflicts or mobbing (below: bad working climate), (II) uncertainty such as short-term employment or looming insolvency (job uncertainty), (III) working under high time pressure and pressure to perform (working under pressure), (IV) overtime or long commuting distances (overtime), (V) a predetermined workflow (low job control), and (VI) shift work or night work (shift work). Data were collected via frequency scales. A stressor “frequently” characterizing the respondent’s daily work is classified as a factual working condition.

The health-related factors comprised risk factors and medical conditions. Data on harmful alcohol consumption were collected using the Alcohol Use Disorder Identification Test–Consumption (AUDIT-C) [54]. Obesity was classified based on statements on height and weight (BMI ≥30). Respondents who reported smoking daily were defined as smokers; those who stated that they were physically active for a maximum of 2 hours weekly were classified as physically inactive. Regarding medical conditions, chronic back pain for at least 3 months and lifelong prevalence of a medically diagnosed chronic condition, namely depression, diabetes, asthma, coronary heart disease, or osteoarthritis, were included. Self-rated health was dichotomized, distinguishing between respondents rating their own health as “very good” or “good” and those rating their health as “moderate” or worse (below: poor self-rated health).

Analyses were restricted to the gainfully employed up to the age of 70 who had not been unemployed during the 12 months prior to the interview (n = 14,041). Twelve-month prevalence of occupational injuries with 95% CI is reported and logistic regression was used for multivariate analyses. The Base Model, which was adjusted for age, gender, work schedule, and years of employment, was followed by a blockwise inclusion of occupation (Model 1), work-related stress (Model 2), and health-related factors (Model 3). Model 4 tested for the association between work-related stress and the occurrence of occupational injuries when controlling for the type of occupation. Model 5 was adjusted for all variables simultaneously. Associations with p-values <0.05 were considered statistically significant. Variance inflation factors (VIFs) were calculated and showed no indication for multicollinearity (mean VIF: 1.26).

Cases were excluded listwise, leading to a final sample of 12,946 respondents across all multivariate models. To find out to what extent respondents who provided complete information are a selective sub-group, we compared their responses with that of respondents with missing data. However, only few differences were found: respondents with complete information were slightly more often men, were somewhat older, reported more frequently to experience physical work-related stress and were slightly healthier with respect to some of the health-related indicators. However, statistical associations were weak since measures of association lay always well below 0.1.

Generally, phone surveys are prone to different forms of selection bias. Therefore, a weighting procedure was applied based on the Behavioral Risk Factor Surveillance System [48], weighting the respondents by sampling probabilities, federal state, gender, age, and education based on the German population on the 31^st^ of August 2008 [55]. A comparison with census data reveals that the sample characteristics are widely consistent with the distribution in the German working population (S1 Table). Differences in the distribution of work schedules can be explained by different approaches to data collection on different forms of minor and short-time employment. Analyses were processed with Complex Samples under IBM SPSS Statistics 20.

## Results

**Fig 1:**
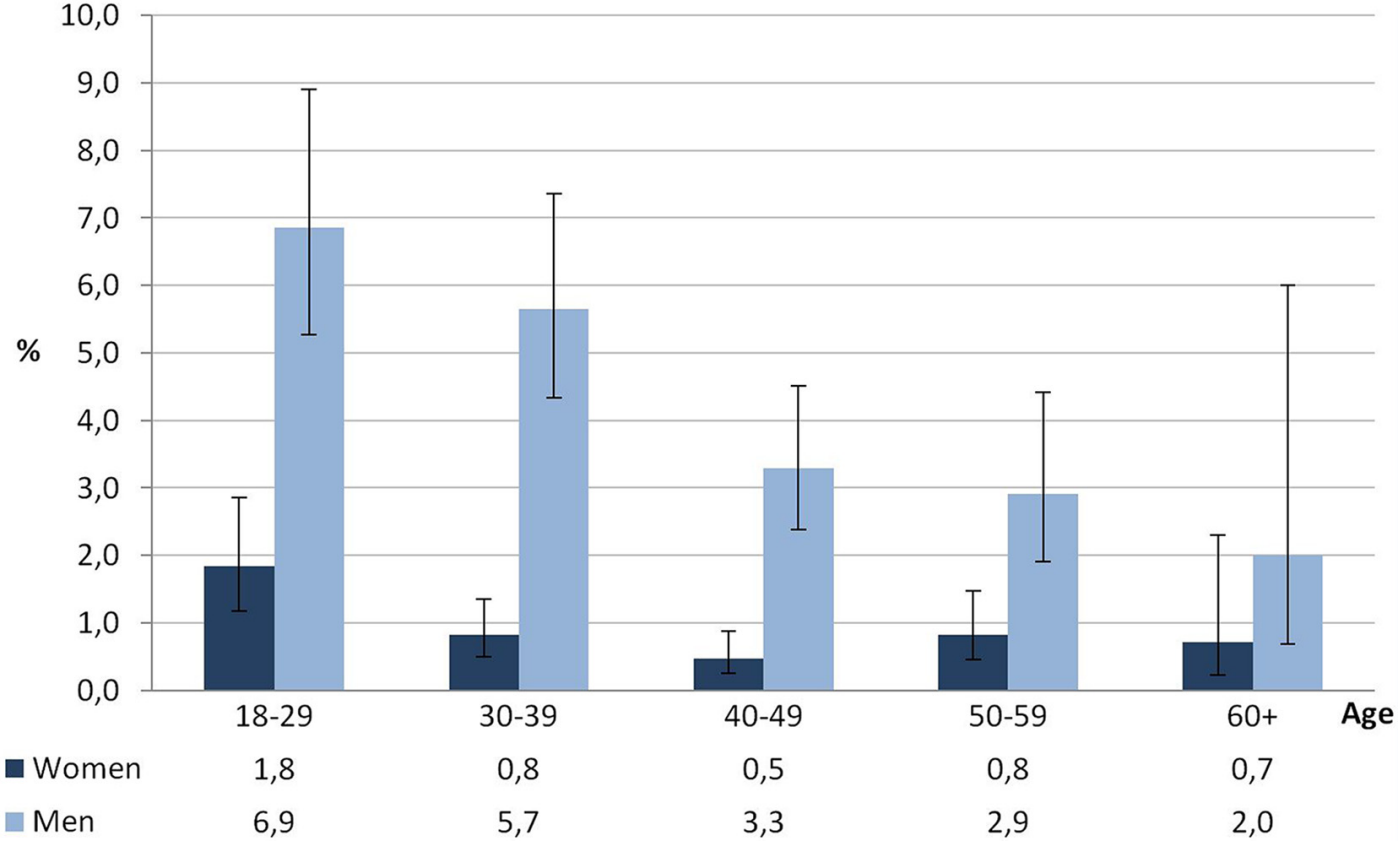
Occupational injuries within the past 12 months among the gainfully employed (age 18–70), GEDA 2010, n = 14,041.

Among the gainfully employed (18–70 years), 2.8% (95% CI 2.4–3.2) suffered at least from one medically treated occupational injury within the preceding 12 months. The prevalence for men amounted to 4.3% (95% CI 3.7–5.0), which clearly exceeds the prevalence for women (0.9%; 95% CI 0.7–1.2). In men, the prevalence of occupational injuries decreases with age. While one in 15 men aged between 18 and 29 suffers a medically treated occupational injury, this falls to one in 50 men aged between 60 and 70. Women aged between 18 and 29 also have a comparatively high chance of occupational injuries. However, beyond the age of 30, the 12-month prevalence in women is less than 1% (Fig 1).

**Fig 2:**
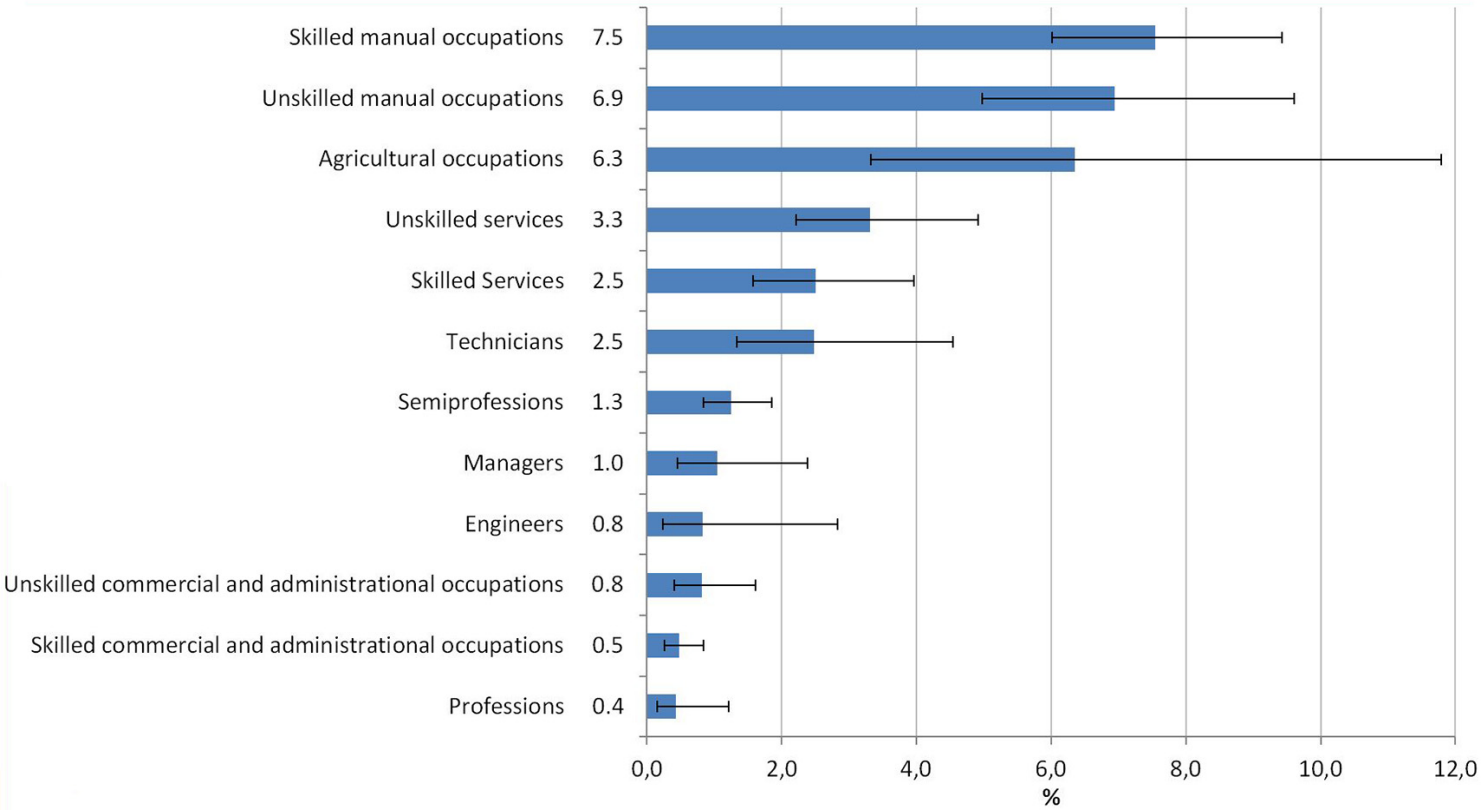
Occupational injuries within the past 12 months by occupational group (age 18–70), GEDA 2010, n = 14,041.

Employees in manual, agricultural, and service occupations, as well as technicians, have higher chances of occupational injuries compared with occupations like clerical work or the professions, where tasks are frequently restricted to desk and screen work (Fig 2). In men, the prevalence of occupational injuries is highest in agricultural (8.9%; 95% CI 4.4–16.9) and unskilled manual occupations (8.9%; 95% CI 6.4–12.3). In women, the highest prevalence is in skilled manual occupations (3.3%; 95% CI 1.4–7.6).

The explanatory power of the multivariate models improves considerably when different blocks of variables are added (Table 2; pseudo R^2^). Specifically, the Base Model (pseudo R^2^: 0.08) shows that men (OR: 4.61; 95% CI: 3.2–6.6) and younger employees (OR 18–29: 1.71; 95% CI: 1.17–2.49) in particular have a high chance of suffering a medically treated occupational injury. In contrast, working hours well below (OR part-time: 0.55; 95% CI: 0.35–0.87) or well above a full-time schedule (OR > 48 h/week: 0.57; 95% CI: 0.37–0.86) are associated with low chances of occupational injury. Taking into account the type of occupation, the model reveals that agricultural, skilled and unskilled manual, and skilled and unskilled service occupations are especially prone to occupational injuries. Furthermore, technicians and members of the so-called semiprofessions also show comparatively high chances of occupational injuries (Table 2: Model 1).

**Table 2:** Associations between occupational injuries and individual factors (blockwise logistic regression; odds ratios), GEDA 2010, n = 12,946.

	n	Model 1	Model 2	Model 3	Model 4	Model 5
**Sex**						
[Ref Women]						
Men	7,522	**3.04**	**4.13**	**4.71**	**3.13**	**3.16**
**Age group**						
[Ref 30–49]						
18–29	2,634	1.38	1.43	**2.07**	1.30	**1.54**
50+	4,088	0.78	0.96	0.66	0.96	0.89
**Work schedule**						
[Ref full-time]						
Part-time	4,787	0.63	0.72	**0.56**	0.78	0.76
> 48h/week	2,011	0.71	**0.48**	**0.54**	**0.51**	**0.49**
**Work experience**						
Years of employment	/	0.988	**0.981**	0.995	**0.978**	**0.979**
**Occupational group**						
[Ref Skilled commercial and administrational occupations]						
Agricultural occupations	244	**11.66**			**5.77**	**5.40**
Engineers	534	1.29			1.32	1.39
Managers	1,204	2.02			1.97	2.07
Professions	741	0.69			0.66	0.75
Technicians	598	**4.39**			**3.37**	**3.41**
Semiprofessions	2,154	**2.89**			1.70	1.75
Unskilled commercial and administrational occupations	1,092	2.43			1.86	1.73
Skilled services	1,294	**5.83**			**4.09**	**4.24**
Skilled manual occupations	1,191	**10.69**			**5.29**	**5.12**
Unskilled services	1,223	**6.07**			**3.38**	**3.13**
Unskilled manual occupations	687	**10.64**			**5.08**	**4.97**
**Physical stress**						
[Ref no]						
Heavy carrying	2,998		**2.10**		**1.81**	**1.78**
Awkward postures	2,944		**1.68**		**1.48**	**1.46**
Environmental stress	3,852		**2.16**		**1.54**	**1.48**
**Psychosocial stress**						
[Ref no]						
Working under pressure	5,889		**1.39**		**1.51**	**1.41**
Overtime	4,972		1.24		1.34	1.34
Shift-work	2,337		1.35		1.34	1.34
Job uncertainty	968		0.67		0.63	0.64
Bad working climate	741		0.67		0.70	0.66
Low job control	2,143		0.94		0.88	0.87
**Behavioral risk factors**						
[Ref no]						
< = 2h physical activity/week	7,000			**1.66**		**1.47**
Smoking daily	3,506			1.35		1.00
Harmful alcohol consumption	4,301			**1.37**		1.34
Obesity	1,651			**1.78**		**1.73**
**Chronic conditions**						
[Ref no]						
Depression	1,869			1.01		1.14
Diabetes	513			**0.15**		**0.18**
Coronary heart disease	298			1.32		1.19
Asthma	1,146			0.98		0.93
Osteoarthritis	1,958			1.30		1.18
Chronic back pain	3,648			**1.43**		1.25
**Self-rated health**						
[Ref (very) good]						
moderate/(very) poor	2,285			1.26		1.14
**R**^**2**^ **Nagelkerke**		0.**14**	**0.16**	**0.11**	**0.18**	**0.19**

Ref: Reference group; Figures in bold: p-value < 0.05; for 95% confidence intervals see S2 Table

Adjusting for the Base Model, of the work-related stressors, physical stress in particular is associated with a higher chance of occupational injury (Table 2: Model 2). Occupations that frequently involve tasks characterized by strenuous effort such as heavy carrying, awkward postures, or environmental stress have a higher prevalence of occupational injuries than occupations that are free of these stressors. Regarding psychosocial stress, this applies to working under pressure, while overtime and shift work show no significant associations.

If health-related factors are added to the Base Model, the odds ratios for occupational injuries are significantly higher among employees with obesity and those who are physically inactive (Table 2: Model 3). The same holds for harmful alcohol consumption and chronic back pain. Unexpectedly, prevalent diabetes is associated with significantly lower chances of occupational injury.

When controlled simultaneously, certain occupations as well as work-related stressors are independently associated with higher chances of suffering occupational injuries (Table 2: Model 4). However, the higher odds ratios attributable to the indicators in both blocks are considerably lower (Table 2: Models 1, 2, and 4).

In the final model, men and young employees still have higher chances of suffering occupational injuries, whereas a regular working schedule of more than 48 hours per week as well as a longer duration of employment are accompanied by lower chances of occupational injury (Table 2: Model 5). Skilled and unskilled manual and service occupations, as well as agricultural and technical occupations, present higher chances of occupational injuries to varying degrees. In addition, the association with occupational injuries of factors such as heavy carrying, awkward postures, environmental stress, and working under pressure persists. Among the health-related factors, lack of physical activity and obesity are still associated with higher chances of occupational injuries in the fully adjusted model whereas diabetes remains negatively associated. In contrast, harmful alcohol consumption, regular smoking, depressions, chronic back pain, coronary heart disease, asthma and osteoarthritis do not show significant associations when the type of occupation and work-related stressors are controlled for.

## Discussion

In Germany, 2.8% of the gainfully employed population suffer at least one medically treated occupational injury per year. Men as well as younger and less experienced employees are especially prone to occupational injuries. However, the main work-related factors are agricultural and manual occupations and physical stress. Among the psychosocial stressors, only working under pressure presents a significantly higher chance of occupational injuries. Of the health-related factors, this holds for obesity and physical inactivity.

Projected to the general population, the prevalence of occupational injuries found in GEDA 2010 rounds up to 1.4 million accident victims per year, although the statutory accident insurance organization reported 1,045,816 occupational injuries in 2010 [56]. The lower figure can be partly explained by the fact that occupational injuries are only notifiable if they lead to sick leave of more than 3 days. Because GEDA 2010 includes occupational injuries irrespective of the number of days absent from work, the two sources are reasonably consistent.

For a detailed interpretation of the presented results, some limitations should be taken into account. First of all, the present analysis is based on self-reported data, which is prone to recall and reporting biases. However, most of the analyzed variables are rather insensitive and generally easy to remember. Even though the study covers a large set of variables, some confounders could be undermining the presented results (e.g. the absence of indicators for relevant factors like fatigue or sleep problems, respectively [35, 57, 58]). Since the study design is cross-sectional, causal statements are difficult. Even though, some correlates (e.g. alcohol use and smoking) generally become prevalent a long time before occupational injuries occur, they might also be one of their consequences. Furthermore, it can't be ruled out that working conditions are partially a result of occupational injuries, underestimating the relevance of work-related factors for the occurrence of occupational injuries. To gain insights into causality, the observed statistical associations should be further examined in longitudinal (e.g. register based) studies. Given that GEDA 2010 is a landline-based telephone survey, certain groups are underrepresented in the sample. These include people who were not at home when the study was conducted (e.g. due to hospitalization) or people who can only be contacted via mobile phones. Because the present analysis is based on survey data, it provides insights from an overall population perspective. However, more task-specific approaches are needed at the occupational level in order to determine the specific meaning of individual risk factors in certain workplace settings. For instance, previous finding have shown that within certain occupations the role of work-related stress or individual risk factors may vary according to age [18, 31, 59]. While such interactions are equally important for the tailoring of preventive measures, they are difficult to address with representative survey data. Furthermore, it is questionable whether the Blossfeld scheme still reflects the present-day occupational structure. However, because new developments such as the IT revolution mainly affected the less accident-prone service sector, the Blossfeld scheme was retained for its particular strengths: that is, it considers country-specific aspects of the labor market, and combines hierarchies of occupational status and vocational training equally in a well-arranged classification. Finally, the small number of occupational injuries sustained by women hinders a meaningful gender-specific analysis. For women, occupational injuries tend to occur in different occupational groups. Thus, the presented results are strongly determined by the occurrence of men’s occupational injuries, while the identification of typical factors for women requires more in-depth research.

The finding that men, as well as younger and less experienced employees, have higher chances of suffering occupational injuries is consistent with the findings of previous studies. In contrast, it is unclear why a regular workload of more than 48 hours per week is associated with lower chances of occupational injury when adjusted for the type of occupation [21, 22]. It might be that within many occupational groups, such a work schedule is associated with higher positions in the organizational hierarchy, which usually involve less hazardous tasks. Moreover, in manual occupations, regular working hours well above the average may be less common in high-income economies with stricter workplace regulations. Thus, the effect of long regular working hours should be analyzed in terms of organizational hierarchies as well as country-specific legislation.

It is equally remarkable that part-time employment only proves to be protective in the base model, while the effect vanishes in models adjusted for occupational groups. Because part-time employment entails shorter periods of exposure to risk, a clear protective effect could be expected. However, a closer look reveals that in GEDA 2010, part-time employment is especially prevalent in less accident-prone but quantitatively important occupations such as the semiprofessions and skilled and unskilled administrative and clerical occupations, where part-time schedules account for about half of all employment. Therefore, it is evident that the protective effect of part-time schedules is largely neutralized when occupations are introduced to the multivariate models. It can be concluded that the lower risk of occupational injuries in such occupational groups may be a consequence not only of less hazardous tasks, but also of shorter exposure periods.

The highest chances of suffering occupational injuries can be found in agricultural and manual occupations. This is confirmed by both official statistics and research findings [5, 6, 13, 16, 23, 56]. The comparatively high chance of suffering occupational injury in the service occupations can be explained by the fact that these occupations involve physically demanding tasks, for example cleaning work, catering, or working in police or fire departments [51]. However, unexpectedly, there are no marked differences in the chances of occupational injury between skilled and unskilled employees, even if physical stress is not statistically controlled. One explanation for this might be that in high-income countries, safety regulations and trends like automation have equalized the relative hazards of skilled and unskilled work.

The physical stressors considered in the present study show clear associations with occupational injuries and are partially independent from the type of occupation. Thus, injury prevention should not be restricted to accident-prone occupations, but should also consider hazardous task profiles that may be present in service or clerical occupations. Furthermore, even if a large part of the burden is associated with the main work-related stressors, especially in agricultural and manual occupations, there is still a much higher chance of occupational injuries in accident-prone occupations in the fully adjusted model.

Among the psychosocial stressors, working under pressure is the only positively associated factor for occupational injuries that proved to be statistically significant. This corresponds with findings supporting an association between workload and occupational injuries [11]. Furthermore, previous research provides little evidence of any association between psychosocial stress and occupational injuries [10, 16]. However, contrary to previous studies, shift work and frequent overtime lack significance in the present analysis [21, 22]. Both associations might be overlaid by working under pressure, which is a stable factor across all models.

Regarding behavioral factors, the present study yields mixed results. There is no significant association between harmful alcohol consumption and occupational injuries. Current research is characterized by conflicting reports, and does not clearly support a positive association [18, 30, 33, 34]. The causal mechanisms that are assumed to explain a possible association between harmful alcohol consumption and the risk of occupational injuries are not confined to problematic drinking behavior on the job. Effects such as hangovers, fatigue, or an underlying disposition toward risk-seeking behaviors are also quoted [33]. Nonetheless, according to the existing literature, drinking on the job can be assumed to be the main mechanism that could be responsible for a possible association between alcohol consumption and occupational injuries. Thus, strict safety regulations that are in effect in many high-income economies like Germany should strongly moderate the association between drinking and occupational injuries and may explain the rather weak association in the present study.

Multiple causal links are also suggested for the association between smoking and occupational injuries [26, 28, 29]. Apart from fire hazards, it is assumed that holding a cigarette may complicate the coordination of manual tasks, and that smoking leads to cognitive impairment, thereby playing a role in the etiology of occupational injuries. Moreover, there is some evidence of worse sleep quality in heavy smokers that could lead to a higher risk of fatigue. However, even though the association between smoking and occupational injuries has been frequently documented [10, 18, 26, 27, 29, 30], it could not be confirmed in the present study. After adjusting for occupation and work-related stress, no visible association remains. Because smoking prevalence is observed along a social gradient, this suggests that the initial association is mainly explained by the respondents’ socioeconomic status, which is reflected in their occupation. Furthermore, as is the case with alcohol consumption, the relevance of regular smoking to the occurrence of occupational injuries may be mitigated by improved safety regulations. Smoking on the job is becoming increasingly restricted in Germany, lowering the chance that the handling of cigarettes on the job plays a role in the etiology of occupational injuries.

In accordance with previous studies, obesity and physical inactivity are clearly associated with a higher chance of occupational injuries in the present analyses [8, 17, 18, 30, 35–37]. Furthermore, these indicators are closely linked to each other. While obesity has adverse effects on motor skills, physical activity helps to prevent obesity and improves body flexibility [17, 36, 37]. Thus, better fitness may enable workers to avoid accidents at work by helping them to either tackle or escape from hazardous situations.

There is some evidence that chronic conditions moderately increase the risk of occupational injuries [14, 38–40]. In part, this evidence relies on large volumes of register-based data, which can easily achieve significance for rather weak associations [40]. Occasionally, it is assumed that this association is caused by generic factors rather than by disease-specific factors. Fatigue caused by medication or a weakened physical constitution may accompany a wide range of chronic conditions [40]. In the present study, the highest odds ratios can be observed for back pain, osteoarthritis, and coronary heart disease, but they lack significance and decrease when work-related stressors and the type of occupation are controlled for.

Moreover, country-specific routines in occupational rehabilitation may moderate the respective injury risks by protecting employees with chronic conditions from hazardous tasks to different degrees. Such a mechanism is supposedly in effect with regard to diabetes. In previous studies, no or weak positive associations were found with occupational injuries [38, 39, 42]. In contrast, in the present analyses, diabetes shows a clearly negative association with the occurrence of occupational injuries. In Germany, it seems to be a common practice to recommend that employees with diabetes quit their occupation if it entails hazardous tasks [41]. This may explain why people with diabetes display a considerably lower chance of occupational injuries in the study-specific context.

## Conclusions

Overall, survey data provide a “bird’s-eye view” of the occurrence of occupational injuries and–from a population perspective–enable the identification of the most relevant injury-related factors, as well as the specification of the main domains for preventive action. According to the results, occupational injury-prevention measures in high-income economies like Germany should focus on certain occupations and physically demanding tasks. Psychosocial stressors like working under high time pressure and pressure to perform, as well as physical fitness, are also important factors. For other psychosocial or health-related factors, the evidence presented does not justify further efforts in relation to the prevention of occupational injuries.

It became clear that specific working conditions like physical or psychosocial stressors are more proximal to the outcome than the type of occupation. While it is true that the type of occupation is strongly associated with the occurrence of occupational injuries, this association wanes, especially when physical stressors are introduced into the analytical models. However, even in the fully adjusted model, the type of occupation remains an important factor. Thus, increased explanatory power could be expected from a further refinement of the range of selected indicators that are more proximal to the outcome than the type of occupation itself: Concerning physical stressors, further research should include a broader range of indicators to better explain the differences between occupational groups.

With respect to psychosocial stressors, the relative meaning of different psychosocial stressors in terms of the etiology of occupational injuries should be further clarified. In the present study, the associations of factors like shift work or overtime seem to be overlaid by different aspects of working under pressure. Working under pressure may be the factor that actually increases the chance of occupational injuries. Thus, to question whether shift work may be irrelevant to the occurrence of occupational injuries in contexts where factors like working under pressure are absent could be one promising research perspective concerning psychosocial stressors.

Little evidence was found for an association between health-related factors and occupational injuries. Future research should place a stronger focus on specific impairments resulting from certain diseases, instead of lifelong prevalence. For instance, research could focus on single mechanisms such as fatigue that may be especially relevant when workers are performing certain tasks or working in specific workplace settings. This could yield a clearer picture concerning higher chances of injury arising from chronic conditions.

Furthermore, factors on higher levels of measurement like companies or nation states could as well help to better explain the occurrence of occupational injuries at individual level. However, companies’ characteristics like safety culture or managerial style are difficult to address with individual level data and would require a multi-level approach of data collection and analysis. The same holds for factors on national level. Health-related factors like smoking or drinking on the job, but also work schedule, shift work, or overtime are framed by national safety regulations. It can be assumed that in countries like Germany with comparatively strict safety regulations, many hazards that emanate from these factors are largely under control. It can be questioned, for instance whether the hazards presented by unskilled work are the same in economies with large service sectors as they are in countries that are still in the process of industrialization. Equally, it can be asked to what extent country-specific safety regulations or the organization of health services moderate the effects of factors such as smoking, drinking, and chronic conditions.

To consider such contextual factors that may constrain or enhance the effect of certain individual factors could be a good starting point for the development of more complex conceptual models for the explanation of occupational injuries. Thus, multi-level approaches including international comparisons should be a promising perspective for future research into occupational injury epidemiology.

## Supporting Information

S1 Table Comparison of sampling characteristics (GEDA 2010, n = 14,041) with census data, gainfully employed men and women (age 18–70). (DOCX)

S2 Table Associations between occupational injuries and individual factors (blockwise logistic regression; 95% confidence intervals of odds ratios), GEDA 2010, n = 12,946. (DOCX)
