# Supplementary material for: Occupational Injuries in Germany: Population-Wide National Survey Data Emphasize the Importance of Work-Related Factors
Source: PLoS One. 2016 Feb 9;11(2):e0148798. doi: 10.1371/journal.pone.0148798 (PMC4747528; doi:10.1371/journal.pone.0148798)
Supplement: S1 Table — (DOCX) [file pone.0148798.s001.docx]

**S1 Table: Comparison of sampling characteristics (GEDA 2010, n=14,041) with census data, gainfully employed men and women (age 18-70)**

|  | **Microcensus 2009^1^**  %_extrapolation_ | **GEDA 2010**  %_weighted_ |
| --- | --- | --- |
| **Sex** |  |  |
| Women | 45.8 | 45.3 |
| Men | 54.2 | 55.7 |
| **Age Group** |  |  |
| 18–29 | 20.5 | 19.9 |
| 30-49 | 51.6 | 51.9 |
| 50+ | 27.9 | 28.2 |
| **Work schedule** |  |  |
| Full-time | 74.4 | 69.0 |
| Part-time | 25.6 | 31.0 |
| **Occupational group** |  |  |
| Skilled commercial and administrational occupations | 20.0 | 18.1 |
| Professions | 4.1 | 3.3 |
| Engineers | 3.5 | 2.8 |
| Managers | 5.9 | 6.8 |
| Unskilled commercial and administrational occupations | 7.4 | 8.7 |
| Semiprofessions | 10.2 | 11.8 |
| Technicians | 4.3 | 4.0 |
| Skilled services | 8.2 | 8.9 |
| Unskilled services | 12.7 | 12.8 |
| Skilled manual occupations | 13.3 | 11.9 |
| Unskilled manual occupations | 8.2 | 8.6 |
| Agricultural occupations | 2.3 | 2.3 |

^1^ Research Data Centres of the Federal Statistical Office and the statistical offices of the Länder, Microcensus 2009, own calculations
