# Supplementary material for: Occupational Injuries in Germany: Population-Wide National Survey Data Emphasize the Importance of Work-Related Factors
Source: PLoS One. 2016 Feb 9;11(2):e0148798. doi: 10.1371/journal.pone.0148798 (PMC4747528; doi:10.1371/journal.pone.0148798)
Supplement: S2 Table — (DOCX) [file pone.0148798.s002.docx]

**S2 Table: Associations between occupational injuries and individual factors (blockwise logistic regression; 95% confidence intervals of odds ratios),**

**GEDA 2010, n=12,946**

|  |  |  | **Model 1** | **Model 2** | **Model 3** | **Model 4** | **Model 5** |
| --- | --- | --- | --- | --- | --- | --- | --- |
| **Sex** | [Ref Women] | Men | 2,08-4,44 | 2,81-6,07 | 3,30-6,74 | 2,12-4,62 | 2,14-4,67 |
| **Age group** | [Ref 30–49] | 18–29 | 0,94-2,03 | 0,97-2,11 | 1,40-3,04 | 0,88-1,93 | 1,02-2,33 |
|  |  | 50+ | 0,48-1,26 | 0,59-1,55 | 0,41-1,09 | 0,60-1,53 | 0,56-1,42 |
| **Work schedule** | [Ref full-time] | Part-time | 0,39-1,01 | 0,45-1,15 | 0,36-0,88 | 0,49-1,25 | 0,48-1,20 |
|  |  | > 48h/week | 0,46-1,11 | 0,31-0,75 | 0,35-0,83 | 0,32-0,81 | 0,31-0,78 |
| **Work experience** |  | Years of employment | 0,971-1,006 | 0,963-1,000 | 0,977-1,013 | 0,960-0,996 | 0,961-0,996 |
| **Occupational group** | [Ref Skilled | Agricultural occupations | 4,61-29,53 |  |  | 2,23-14,95 | 2,13-13,68 |
|  | commercial and | Engineers | 0,33-5,14 |  |  | 0,34-5,09 | 0,36-5,39 |
|  | administrational | Managers | 0,71-5,78 |  |  | 0,70-5,59 | 0,74-5,80 |
|  | occupations] | Professions | 0,18-2,63 |  |  | 0,17-2,53 | 0,19-2,92 |
|  |  | Technicians | 1,86-10,33 |  |  | 1,42-8,04 | 1,42-8,18 |
|  |  | Semiprofessions | 1,40-5,97 |  |  | 0,79-3,68 | 0,81-3,80 |
|  |  | Unskilled commercial and administrational occupations | 0,98-6,00 |  |  | 0,75-4,61 | 0,70-4,32 |
|  |  | Skilled services | 2,74-12,40 |  |  | 1,88-8,90 | 1,95-9,20 |
|  |  | Skilled manual occupations | 5,65-20,23 |  |  | 2,67-10,49 | 2,57-10,19 |
|  |  | Unskilled services | 3,01-12,23 |  |  | 1,59-7,18 | 1,46-6,73 |
|  |  | Unskilled manual occupations | 5,38-21,02 |  |  | 2,42-10,65 | 2,37-10,46 |
| **Physical stress** | [Ref no] | Heavy carrying |  | 1,43-3,07 |  | 1,24-2,63 | 1,22-2,61 |
|  |  | Awkward postures |  | 1,18-2,39 |  | 1,05-2,09 | 1,03-2,07 |
|  |  | Environmental stress |  | 1,53-3,06 |  | 1,07-2,23 | 1,02-2,15 |
| **Psychosocial stress** | [Ref no] | Working under pressure |  | 1,02-1,90 |  | 1,10-2,07 | 1,03-1,92 |
|  |  | Overtime |  | 0,89-1,73 |  | 0,95-2,07 | 0,96-1,89 |
|  |  | Shift-work |  | 0,98-1,87 |  | 0,94-1,87 | 0,94-1,91 |
|  |  | Job uncertainty |  | 0,39-1,18 |  | 0,36-1,10 | 0,37-1,13 |
|  |  | Bad working climate |  | 0,37-1,22 |  | 0,38-1,28 | 0,36-1,21 |
|  |  | Low job control |  | 0,65-1,38 |  | 0,61-1,28 | 0,60-1,28 |
| **Behavioral risk** | [Ref no] | <=2h physical activity/week |  |  | 1,21-2,26 |  | 1,07-2,02 |
| **factors** |  | Smoking daily |  |  | 0,98-1,84 |  | 0,72-1,39 |
|  |  | Harmful alcohol consumption |  |  | 1,01-1,85 |  | 0,99-1,83 |
|  |  | Obesity |  |  | 1,21-2,59 |  | 1,18-2,54 |
| **Chronic conditions** | [Ref no] | Depression |  |  | 0,61-1,68 |  | 0,66-1,96 |
|  |  | Diabetes |  |  | 0,04-0,59 |  | 0,04-0,75 |
|  |  | Coronary heart disease |  |  | 0,48-3,74 |  | 0,39-3,70 |
|  |  | Asthma |  |  | 0,60-1,61 |  | 0,56-1,54 |
|  |  | Osteoarthritis |  |  | 0,83-2,03 |  | 0,74-1,88 |
|  |  | Chronic back pain |  |  | 1,01-2,03 |  | 0,88-1,78 |
| **Self-rated health** | [Ref (very) good] | moderate/(very) poor |  |  | 0,84-1,91 |  | 0,75-1,73 |
|  |  |  |  |  |  |  |  |
